# Creatine Supplementation in Children and Adolescents

**DOI:** 10.3390/nu13020664

**Published:** 2021-02-18

**Authors:** Andrew R. Jagim, Chad M. Kerksick

**Affiliations:** 1 Sports Medicine, Mayo Clinic Health System, La Crosse, WI 54601, USA; ckerksick@lindenwood.edu; 2 Exercise & Sport Science Department, University of Wisconsin-La Crosse, La Crosse, WI 54601, USA; 3 Exercise & Performance Nutrition Laboratory, Lindenwood University, St. Charles, MO 63301, USA

**Keywords:** ergogenic aid, dietary supplement, youth, athletes

## Abstract

Creatine is a popular ergogenic aid among athletic populations with consistent evidence indicating that creatine supplementation also continues to be commonly used among adolescent populations. In addition, the evidence base supporting the therapeutic benefits of creatine supplementation for a plethora of clinical applications in both adults and children continues to grow. Among pediatric populations, a strong rationale exists for creatine to afford therapeutic benefits pertaining to multiple neuromuscular and metabolic disorders, with preliminary evidence for other subsets of clinical populations as well. Despite the strong evidence supporting the efficacy and safety of creatine supplementation among adult populations, less is known as to whether similar physiological benefits extend to children and adolescent populations, and in particular those adolescent populations who are regularly participating in high-intensity exercise training. While limited in scope, studies involving creatine supplementation and exercise performance in adolescent athletes generally report improvements in several ergogenic outcomes with limited evidence of ergolytic properties and consistent reports indicating no adverse events associated with supplementation. The purpose of this article is to summarize the rationale, prevalence of use, performance benefits, clinical applications, and safety of creatine use in children and adolescents.

## 1. Introduction

Creatine (methyl-guanidine-acetic acid) is a naturally occurring amino acid-like compound that is endogenously produced within the human body and exogenously consumed in food sources such as red meat and seafood [1]. Creatine is primarily stored within skeletal muscle tissue (~95% of total stores) mostly in the form of phosphocreatine, which functions as an energy source through an enzymatic reaction involving creatine kinase, phosphocreatine, and adenosine di-phosphate to yield adenosine tri-phosphate (ATP). The inorganic phosphate and free energy yielded from ATP hydrolysis is then used for cellular work, with increasing demands as the intensity of effort is increased [1]. Research has indicated that internal phosphocreatine stores can be increased by 15–40% through creatine supplementation strategies, which can subsequently have various performance-related benefits in isolation, and when used in conjunction with structured exercise training programs over time [2,3,4,5,6]. The purported ergogenic benefits of creatine are well-supported within the literature for multiple populations [1]. Because of the strong evidence for ergogenic benefits pertaining to high-intensity exercise performance, as well as an increase in strength and skeletal muscle hypertrophy, creatine is a popular dietary supplement of choice among athletic populations. A systematic review by Knapik et al. [7] in 2016 reported creatine use in 50 out of 159 (31%) unique studies examining the prevalence of dietary supplement use by athletes of all ages. Creatine is not only one of the more popular dietary supplements from a performance perspective, but there is also strong evidence to support its use in clinical settings for a variety of patient populations [1,7]. However, the use of creatine among children and adolescent populations still remains somewhat controversial. While the physiological rationale regarding an ergogenic benefit in adolescents is similar to that seen in adults [8], the lack of randomized controlled trials and clinical data supporting the safety of creatine supplementation protocols among adolescent populations has resulted in hesitation regarding its widespread recommendation by some practitioners. Despite these concerns, creatine is still a popular dietary supplement of choice among adolescent populations and has been studied for its ergogenic potential in select athletic populations, albeit mostly in international (non-US) settings. It is clear that more research is warranted to better understand the short and long-term safety of creatine among adolescent athletes, however there is a precedent for its use among certain adolescent populations, both in athletes and special populations. Therefore, the focus of this article is to summarize the prevalence of use, performance benefits, clinical applications, and safety of creatine use in adolescents. For the purposes of the current review, children are defined as individuals between the ages of 0–12 years, while adolescents are defined as individuals between the ages of 13–19 years of age.

## 2. Effects of Creatine Supplementation on Creatine Content

Although limited in number, select studies have demonstrated that creatine supplementation is an effective nutritional strategy to promote increases in the phosphocreatine content and energy status of the cell among pediatric populations [9,10]; however, some evidence suggests this is potentially so, but to a lesser extent when compared to what is commonly reported in adult populations [1]. Depending on baseline levels of creatine content, which tend to be heavily influenced by exogenous creatine intake [11], increases of 10–40% in creatine or phosphocreatine content within skeletal muscle tissue are routinely reported following periods of creatine supplementation in adult populations [1]. Interestingly, preliminary evidence suggests that an age-dependent effect of creatine supplementation may exist regarding intramuscular creatine uptake, thereby indicating that the development of age-specific supplementation strategies may be warranted [9,10]. Further, research in adult populations [3,12,13] has indicated a moderate degree of variability in tissue uptake response to creatine supplementation protocols. It is currently unknown if similar variabilities (i.e., responder vs. nonresponder effects) are also present among children and adolescents. Due to age and ethical considerations, the majority of research in pediatric populations has relied on magnetic resonance imaging or laboratory markers as indirect measures of creatine and phosphocreatine content rather than through muscle biopsies, which is a common technique used for measuring intramuscular creatine content in adult populations. Additionally, lower doses of creatine are sometimes used among pediatric populations, which is also likely to influence the magnitude of changes observed in creatine content following a supplementation period. Therefore, it is difficult to directly compare the efficacy of creatine supplementation strategies between age groups when different techniques are used to quantify creatine content and different dosing strategies may be employed. However, a study by Solis et al. [10] was able to examine changes in brain and muscle phosphocreatine content across three age groups (children, *n* = 15, adult omnivores, *n* = 17, adult vegetarians, *n* = 14, and elderly adults, *n* = 18) using ^31^P-magnetic resonance spectroscopy, following a standard creatine loading protocol (0.3 g/kg/day for 7 days). Results indicated intramuscular phosphocreatine content significantly increased by 13.9% while brain phosphocreatine levels only increased by 2.1% among the group of children [10]. Comparatively, following the same creatine dosing regimen, larger increases in muscle phosphocreatine content were observed in the elderly (22.7%) but not adult omnivores (10.3%) when compared to the children. It is also worth noting that lower baseline levels of muscle phosphocreatine content were observed in the children compared to the adult groups. Using a lower dose (5 g/day), but over an 8-week period, Banerjee et al. [9] reported significantly greater increases in the mean phosphocreatine/inorganic phosphate ratio following creatine supplementation compared to placebo (Creatine: 4.7; 95% CI: 3.9–5.6 vs. Placebo 3.3; 95% CI 2.5–4.2; *p* = 0.03) in patients with Duchenne Muscular Dystrophy. Alternatively, contradictory reports to these outcomes regarding the efficacy of creatine supplementation have indicated little to no impact on intramuscular phosphocreatine content; however the relative dose used in these studies may have been too low (0.1 g/kg/day for 12 weeks) to elicit any meaningful changes in creatine content [12,13]. Additionally, it is also worth noting these studies were conducted in patients with childhood-onset systemic lupus erythematosus and juvenile dermatomyositis, which may have influenced the efficacy of creatine supplementation in its ability to increase phosphocreatine content. Preliminary evidence among specialized clinical populations with inborn errors of metabolism, such as creatine deficiencies, has also indicated that creatine supplementation can positively influence brain creatine content with a subsequent influence on cognition, natural development, and quality of life [14,15,16]. However, as highlighted previously by Solis et al. [10], it was reported that a standard creatine loading regimen (0.3 g/kg/day for 7 days) in prepubescent children, omnivore adults, vegetarian adults, and elderly adults was not able to elicit significant changes in brain phosphocreatine content [10]. Similar findings have also been observed in healthy young children between the ages of 10 to 12 years of age [17], which failed to observe significant increases in brain creatine content following creatine supplementation. However, a review by Dolan et al. [18] highlighted multiple studies in adult populations, all of which utilized varying creatine supplementation strategies (2–20 g per day from 5 days to 8 weeks), that were able to demonstrate significant increases in brain creatine and phosphocreatine content following supplementation. It is possible that higher doses of creatine over longer periods of time may be required for meaningful increases in brain creatine content to occur among healthy populations as has been previously suggested [18]. The potential increase in brain creatine content following supplementation may also be age-dependent. Nevertheless, more research is needed to identify the extent to which creatine supplementation can impact brain creatine levels and whether or not modified creatine supplementation strategies for this purpose are warranted.

## 3. Prevalence of Use among Adolescents

The popularity of dietary supplements among adolescent populations has slowly increased over the past two decades. One of the more popular self-reported dietary supplements used among this population is creatine, with evidence of use for performance-enhancing purposes beginning in the late 1990s [7]. In 2001, when 674 high school athletes within the United States were surveyed on the use and perceptions of oral creatine supplementation, results indicated that 75% of athletes were aware of creatine and 16% reported some level of previous use [19]. Males exhibited much higher (23%) prevalence rates of use when compared to females (2%), with the percentage of creatine use increasing with grade level as 5% of 9th grade survey respondents reported creatine use compared to 22% of those in 12th grade [19]. Interestingly, 97% of athletes indicating creatine use reported a benefit from creatine, while 26% reported side-effects, a value that is far above the typical rate of side-effects from creatine supplementation within the literature [1]. It is possible that underlying confusion or bias regarding the side-effects of creatine supplementation influenced the high rate of side-effects reported in this study. Nevertheless, a more in-depth discussion regarding adverse events and the safety of creatine supplementation can be found later in the review in Section 6. Nearly 70% of creatine users reported ingesting a loading dose (i.e., 5–10 g for a period of 3–5 days) and nearly all creatine users reported the use of a maintenance dose following the loading period. In 2002, 16.7% of 4011 high school student-athletes surveyed from the United States self-reported current or prior creatine use with a higher use rate among males (25.3%) compared to females (3.9%) [20]. Additionally, the authors noted a large range of creatine use across sport-type, with football representing the highest (30.1%) rates of use and female cross-country representing the lowest (1.3%) rates of use. Increased strength was indicated as the most common reason for creatine supplementation. In 2004, among a convenience sample of 333 adolescents in a Midwestern Canadian province, 5.3% of those surveyed self-reported prior creatine use with 6.6% self-reporting they would use creatine in the future [21]. Individuals who self-reported current creatine use spent more time being physically active throughout the week compared with those not reporting creatine use. Additionally, among this population a higher rate of use was observed in males compared to females, which is in accordance with previous findings. Interestingly, 43.1% of athletes did not know if creatine would enhance performance while 14.2% believed that it would not improve performance, which highlights the need for improved education in this area. Shortly thereafter, in 2008, Hoffman et al. [22] surveyed a group of 3248 students in grades 8–12 within the United States and found 7% of survey respondents self-reported creatine use. Boys reported greater use of creatine with progressively increasing rates of creatine use reported at higher grade levels (22% of 12th grade boys). When asked about their primary source of information regarding dietary supplements, teachers (36%) and parents (16%) were the most common responses among all students. In 2012, results from a large subset (*n* = 9417) of the National Health Interview Survey respondents indicated that 34.1% of the children or adolescents (mean age = ~11 years) reporting the use of dietary supplements to enhance sports performance, self-reported using creatine [23]. Most recently, in 2020 among a sample of Australian adolescent boys participating in a variety of sports, 8.4% of survey respondents reported current use of creatine and 25.7% reported the intent to use creatine or other dietary supplements in the near future [24]. Drive for muscularity, participating in weight training, and sport participation were strong predictors of supplement use [24]. It is important to note that there is an underlying concern that creatine use among adolescents may be a predictor of future illegal performance enhancing substance use. However, the survey utilized to examine this relationship [25], inappropriately categorized creatine as an androgenic anabolic agent, rather than an amino-acid compound, thereby introducing bias to the future projection and therefore caution should be used when interpreting these findings.

The prevalence of creatine use tends to be higher among international adolescent athletes competing at the elite level. For example, Petroczi et al. [26] surveyed elite adolescent athletes as part of the United Kingdom Sport 2005 Drug Free Survey and when a subsample of 874 athletes were reanalyzed, 36.1% of athletes reported using creatine. Of note, a strong relationship was present between reasons for creatine use and physiological rationale among creatine users. This relationship indicates that those self-reporting creatine use were able to correctly identify the purported benefits while selecting physiologically appropriate reasons for use, which was not observed for other dietary supplements. Similarly, in 2008 Petroczi and Naughton [27] surveyed a cohort of 403 elite athletes from the United Kingdom and reported 28% of athletes self-reported creatine use. More recently in 2019, Jovanov et al. [28] surveyed 348 male and female adolescent athletes across four different countries who were all competing at an international level for their respective countries and reported that 25.3% of all athletes indicated using creatine. In alignment with previous studies, a significantly higher proportion of male athletes reported using creatine compared to female athletes (72.0% vs. 28.0%). Additionally, a higher proportion of athletes in the 17–18 year old age group reported creatine use, compared to the 15–16 year old group (60.0% vs. 40.0%). It is important to highlight these findings as they are some of the highest creatine use rates reported among a cohort of adolescents, which may be an indication of recent trends for higher creatine use. These outcomes may also be attributed to the continued growth of the dietary supplement market, popularity of competitive youth sports at an elite level, and the increasing reputation of creatine’s ergogenic properties. It is also important to note that there has been a significant increase in the prevalence of creatine supplementation among female athletes, in particular, over the past 20 years [19,28]. While creatine appears to be a popular dietary supplement of choice among elite adolescent athletes, creatine is still less frequently used when compared to other dietary supplement categories such as multivitamins, protein powders, and energy products, in which usage rates of these product categories can range anywhere from 60–80% among adolescent athletes [26,27,28,29,30,31,32]. More research is needed to better understand the contextual factors underpinning these trends in dietary supplement use among adolescents.

## 4. Performance Benefits

When compared to adults, a limited number of controlled investigations examining the ability of creatine supplementation to impact measures of exercise performance among adolescent populations exist. All available studies to date, have been completed in only two types of athletes: swimming (*n* = 5) and soccer (*n* = 4). Additionally, studies were completed across all parts of the globe with two studies being completed in Brazil and one study being completed in Hungary, Australia, USA, United Kingdom, Iran, and Yugoslavia. The studies completed on swimmers differed somewhat in the dosing regimen that was employed. Three of the studies utilized a loading phase for the entirety of the supplementation regimen, incorporating doses of 21 g per day for nine days, 20 g per day for five days, and 20 g per day for 4 days [33,34,35]. The other two studies used a combination of a loading (5 days at 20 g/day or four days at 25 g/day) and a maintenance phase (5 g/day for 22 days or 5 g/day for 2 months) [36,37]. Various parameters of swimming performance were assessed ranging from in-water sprint swimming performance to power outputs completed during a swim bench/ergometer test. All studies that reported outcomes within the initial 4–9 days of supplementation identified an improvement in various performance measures such as swim bench test performance, sprint swimming performance, dynamic strength, and anaerobic exercise performance. The longest study by Theodorou et al. [37], reported improvements in interval swimming exercise performance after the loading phase, but no further improvement after a maintenance dose was administered. Alternatively, Dawson et al. [36] reported improvements in swim bench performance, but not sprint swimming performance after completion of both a loading and maintenance phase. While not directly performance related, Juhasz et al. [38] indicated that creatine supplementation may also be an effective strategy to support the rehabilitation of overuse-associated tendinitis in adolescent swimmers when combined with a targeted physical therapy program. Notably, when indicated by the authors, no adverse events were reported in any of these studies.

The studies that enrolled adolescent soccer athletes as study participants ranged in duration from 7–49 days. Three studies were seven days in duration and employed loading phases that each delivered different loading doses (0.03 g/kg/day, 20 g/day, and 30 g/day) [39,40,41]. One study [42] was seven weeks in duration and used a seven-day loading phase (20 g/day) followed by a six-week maintenance dose of 5 g/day. All studies were placebo-controlled. Of interest, all three studies that were <7 days in duration, reported statistically significant improvements in various performance outcomes. For example, Mohebbi et al. [39] reported a significant improvement in repeat sprinting and soccer dribbling performance, while Ostojic et al. [40] reported improvements in performance of a soccer dribbling test, countermovement jump, and power production during a sprint. Lastly, Yanez-Silva et al. [41] reported improvements in peak and mean power output as well as total work completed during a Wingate anaerobic capacity test. The remaining study, Claudino et al. [42] failed to report any improvements in lower body power production after 14 male adolescent soccer athletes completed a one-week loading and a six-week maintenance dose phase. Finally, and similar to what was observed with the studies involving swimming, creatine was well tolerated with no adverse events being reported. A summary table of these studies has been included in Table 1.

In summary, limited research is available that has examined the potential of creatine supplementation to impact various aspects of exercise performance. Of the limited work that has been completed, creatine appears to be well-tolerated with no adverse events being reported and consistent improvements in assessments associated with swimming and soccer performance observed in adolescent athletes. Future research is warranted to better evaluate the ability of creatine to influence other types of exercise performance and sport-specific activities as well as the potential for synergistic adaptations to exercise training. Moreover, additional research is urgently needed in adolescent females across all sport types and while research is welcomed in swimming and soccer, future research should examine the potential of creatine in other popular team-based sports where strength and power are key physiological attributes in predicting sporting success.

## 5. Clinical Applications

Over the past 30 years, the discovery of inborn errors of metabolism and the potential physiological, neurological, and neuroprotective benefits of creatine have led to advancements in the therapeutic use of creatine. As such, several pediatric clinical populations have been shown to benefit from creatine supplementation, which notably includes patients with genetic defects associated with creatine deficiency. Table 2 presents a summary of studies that have examined the therapeutic benefits of creatine supplementation for a variety of clinical disorders. To date, the majority of clinical trials investigating the therapeutic potential of creatine supplementation in pediatric populations have focused on creatine (and/or creatine transporter) deficiencies, inborn errors of metabolism, neuromuscular disorders, and myopathies [9,44,45,46,47,48,49]. Guanidinoacetate methyltransferase (GAMT) and arginine:glycine amidinotransferase (AGAT) deficiency, are types of inborn errors of creatine metabolism, collectively characterized as cerebral creatine synthesis deficiencies [47,50]. Several studies and case reports have indicated that creatine supplementation can restore tissue creatine content and improve some of the symptoms resulting from creatine deficiencies [51,52,53,54]. Since its discovery in 1994, GAMT deficiency has shown to be treatable through creatine supplementation strategies [51,53]. For example, in 1996, Stockler et al. [54] treated an infant patient with GAMT deficiency using a creatine replacement therapy of 4–8 g/day over a 25-month period and reported substantial clinical improvement, normalization of brain MRI abnormalities, and improvements in electroencephalogram readings post-treatment. Since that time, several additional case reports and reviews have been published, highlighting effective strategies to diagnose and treat cerebral creatine deficiency with consistent improvements in intellectual development reported, especially when early detection and ensuing treatment were employed [51,52]. While similar in nature, AGAT deficiency is extremely rare with only 20 documented cases worldwide [52]. AGAT deficiency is also an autosomal recessive disorder that disrupts the biosynthesis of creatine and is associated with a variety of clinical features such as intellectual development disorder, speech delays, autistics behaviors, and occasional seizures [52]. Thankfully, AGAT also appears to betreatable with creatine supplementation. For example, Ndika et al. [14] treated a 9-year-old female pediatric patient with AGAT deficiency with up to 800 mg/kg/day of creatine over an 8-year period and reported partial recovery of cerebral creatine levels with the patient demonstrating superior nonverbal and academic abilities at age 9, compared to initially presenting with a score of 43% of her chronological age at 16 months when assessed using the Bayley’s Infant Development Scale. Although similar, creatine transporter deficiency is another inborn error of metabolism that can result in creatine deficiencies in select tissues, particularly within the brain [51]. Creatine transporters are membrane-bound transport proteins that have been found in a variety of different tissues and are required for tissue uptake of creatine against its concentration gradient. Moreover, creatine transporter 1 (CrT1) is expressed ubiquitously across human tissues and deficiencies of this protein are another type of creatine metabolism disorder that can result in brain atrophy, intellectual disabilities, and developmental delays [51]. However, this defect is not as responsive to exogenous creatine supplementation strategies, as the deficiency is attributable to an inability to transport creatine across the cell membrane, rather than a lack of creatine availability [47]. As such, current research has focused on identifying alternative strategies that may enhance brain creatine content in these populations. For example, recent work has demonstrated early promise with the use of creatine fatty esters and lipid nanocapsules as a nutrition-based therapeutic treatment for creatine transporter deficiency [55,56]. These creatine esters and lipid based nanocapsules are better able to cross the blood−brain barrier, thereby helping to increase brain creatine content and correct the creatine deficiency [56].

Considerable research has also been dedicated to investigating the therapeutic benefit of creatine in the management of myopathies. As a high percentage of creatine is stored within skeletal muscle tissue, muscle disorder pathologies, such as myopathies, are often associated with reduced intramuscular concentrations of creatine, phosphocreatine, and ATP in addition to subsequent neuromuscular impairments and muscle weakness [66]. As such, a strong underlying physiological rationale exists to support the potential of creatine supplementation as a therapeutic agent in the management of myopathies. For example, Duchenne’s muscular dystrophy is one such myopathy that is progressive in nature with no known cure. Patients are often prescribed corticosteroids to slow disease progression, which can have several adverse side effects when used long-term. Because of the catabolic nature of corticosteroid therapy, and musculoskeletal pathology associated with muscular dystrophy, creatine supplementation has been identified as a therapeutic agent to potentially counteract the deleterious effects of both the disease, and comorbidities which arise secondary to the primary corticosteroid treatment. Favorable improvements have been observed for fat-free mass and strength in pediatric patients [45]. A major challenge with clinical trials investigating the therapeutic benefits of creatine supplementation in patients with various types of myopathies is dealing with the heterogeneity of the disease itself. The diversity in how the disease manifests in patients can subsequently influence the time course of disease progression and individualistic nature of active versus remission disease states, all of which can be difficult to account for with an optimal study design.

Gyrate atrophy of the retina and choroid is another creatine deficiency disorder that is characterized as an enzymatic disorder attributable to defects in ornithine aminotransferase, resulting in elevated levels of ornithine, which negatively impacts creatine synthesis [57,67]. As a result, creatine concentrations in serum, urine, erythrocytes, brain, and muscle are reduced in these patient populations [58,67]. These patients present primarily with eyesight problems beginning as early as age five, which progressively deteriorate over time. Results from studies spanning up to 72 months in this patient population indicate that creatine supplementation can slow disease progression while also helping to maintain levels of type II skeletal muscle fiber content [68,69].

Lower amounts of daily exogenous creatine intake have also been associated with depression in young adult populations as Bakian et al. [70] observed a significantly higher prevalence of depression (10.2/100 persons) in the lowest quartile of dietary creatine intake compared to the highest quartile of creatine intake, which had a depression prevalence rate of 6.0/100 persons. This relationship appeared to be strongest in females and those in the 20–39 years of age category and therefore may also extend to adolescents. Additionally, early evidence indicates that creatine may be used as an adjunctive therapy in the actual management of clinical depression [71,72,73]. Creatine supplementation has also been used as an experimental therapeutic agent for conditions pertaining to hypoxia and energy-related brain pathologies such as traumatic brain injuries or cerebral ischemia in pediatric patients [20,49,74]. There has also been recent interest in examining the potential benefits of creatine supplementation for pregnant women with potential benefits extending to the developing fetus [75,76]. Currently, clinical trials are underway to better understand how creatine may affect both the mother and developing fetus [75]. It is also worth noting that a growing body of evidence exists demonstrating that creatine may also confer a variety of physiological benefits for multiple clinical conditions in adult populations, such as mitochondrial disease, neurological disorders, and autoimmune disorders [47,74], but the extent to which these findings may extend to pediatric populations requires more research due to the limited data currently available. Lastly, and a point that is beyond the scope of this review, all of these findings may hold particular importance for any clinical population (adult or adolescent) who are vegetarians as they may be susceptible to low daily creatine intake through diet alone, as has been reported in adults [3,11,61].

## 6. Safety

An extensive summary highlighting the safety of creatine supplementation was recently addressed in a 2017 Position Stand by Kreider et al. [1]. However, to date, all published studies involving an a priori research question and utilizing a study designed to examine the safety of creatine supplementation have thus far only been completed in adult populations. Currently, no studies have been published examining safety considerations in healthy young or adolescent athletic populations. Importantly and practically speaking, no indications currently exist as to why a similar safety profile would not be observed in adolescents, and as highlighted throughout the previous sections, multiple creatine supplementation studies have been conducted in adolescent athlete and clinical populations with no adverse events reported. Several of these studies have even closely monitored laboratory markers throughout the supplementation period with no indications of clinically relevant adverse effects observed. While the authors agree, and would like to publicly state that an absence of self-reported adverse events by study subjects is not a confirmation of safety, it does support the hypothesis that creatine is likely safe for this population. Nonetheless, safety studies in youth and adolescent populations using randomized controlled trial designs are desperately needed to help continue building the safety profile for creatine supplementation among these younger age groups. Another way to assess safety-related concerns is to examine adverse event reports that are submitted to the Center for Adverse Event Reports, which is overseen by the Center for Food Safety and Applied Nutrition as part of the United States Food and Drug Administration (FDA). When searching this adverse event reporting system database, which is publicly available, only 22 out of the 15,274 (0.144%) adverse events reports were associated with creatine during the 2018–2020 reporting period, after filtering out food, cosmetics, and adverse events without a known product code (Accessed 5 February 2021; file dates January 2018–March 2020) [77]. In perhaps the strongest testament to the safety of creatine, the United States FDA recently designated creatine as “generally recognized as safe” (GRAS) (https://www.fda.gov/media/143525/download) [78]. Ultimately, this classification indicates that creatine is considered safe under the conditions of its intended use based on the currently available scientific evidence as decided upon by a panel of qualified content experts. Importantly, and a point that is pertinent to the topic of the current article, this designation of safety extends to older children and adolescents.

A unique 2019 study published by Simpson and colleagues [79] may provide some of the first published data involving youth athletes following creatine supplementation with outcomes pertaining to safety implications, and adverse events. In this study 19 elite soccer players (*n* = 13, U18 “Under 18 years” and *n* = 6, U21 “Under 21 years”) completed an eight-week supplementation regimen of creatine monohydrate (0.3 g/kg/day for 7 days and 5 g/day for the remaining seven weeks) in a randomized, double-blind, placebo-controlled fashion. Before and after supplementation, study participants had airway inflammation (using exhaled nitric oxide) and airway responsiveness to dry air (hyperpnea) assessed before and after supplementation. Participants with previous pulmonary disease were excluded and all participants were assessed for unknown or undiagnosed allergies prior to the study. A statistically meaningful trend (*p* = 0.056) between the groups with medium to large observed effect sizes (*n* = 0.199) were found regarding the amount of exhaled nitric oxide (an assessment of airway inflammation). There was also as a trend (*p* = 0.070, *n* = 0.975) of forced expired volumes in one second to reduce with creatine supplementation when compared to placebo. The authors concluded that, “we cannot exclude that creatine supplementation has an adverse effect of the airways of elite athletes, particularly in those with allergy sensitization.” These isolated and initial findings are some of the first published data to suggest that creatine supplementation may compromise airway health and thus more research is needed to confirm or refute these findings.

## 7. Practical Recommendations and Future Directions

In conclusion, there appears to be strong evidence of creatine use among adolescents, particularly among male athletes with the highest usage rates evident among international adolescent athletes competing at the elite level. The majority of adolescents who self-report using creatine, appear to get their information from friends, coaches, and parents. However, the need for replication of dietary supplement questionnaire studies continues to be present because of the recent growth in the dietary supplement industry. Additionally, the recent popularity of marketing through social media platforms and online markets for supplement companies has likely altered how adolescents perceive and obtain information surrounding dietary supplements in addition to how they purchase them. Further, there continues to be a need for more education regarding safe and effective dietary supplement strategies among adolescents, rather than strict policies avoiding or advocating against their use. Even young adults pursuing careers in health professions do not appear to have sufficient knowledge of the safety, regulation, and efficacy of various dietary supplements [80], which reinforces the need for more education and dialogue surrounding the topic, particularly when one considers the high prevalence of use across all populations.

A small number of investigations have reported on the ergogenic benefits of creatine supplementation in adolescent athletes (Table 1), with the majority of this data being published in international (non-US) adolescent males competing in swimming or soccer. However, a paucity of observational and no experimental research exists that examines changes in the clinical health markers among healthy adolescents who are supplementing with creatine, especially those who regularly partake in high-intensity exercise training and athletic competition. While data is limited, it is not entirely absent as many studies report on the lack of reported side-effects, nonsignificant changes in laboratory markers of kidney and liver function, and a lack of changes in inflammatory cytokines among clinical populations, therein supporting the hypothesis that creatine supplementation is likely safe for an adolescent population. The lack of adverse event reports in the literature among clinical populations is telling, particularly when considering that several of the patients are on immunosuppressive therapies or have multiple comorbidities that could negatively influence various indictors of health status. Regardless, a dire need exists for prospective randomized, double-blind, placebo-controlled trials examining the safety and efficacy of creatine among children and adolescent populations; both among athletes and the general population. Equally important are well-powered, randomly controlled trials examining the status and changes in body water and cellular hydration status before, throughout, and after standard regimens of creatine supplementation and to what extent these changes impact creatine content, performance and physiological adaptations to regular exercise training. Priority should first be placed on examining the effects of various creatine-dosing strategies on markers of clinical health or any contraindications for use among this population. From there, emphasis should be placed on continuing to explore the potential benefits of creatine among clinical populations where there might be a unique physiological rationale for a therapeutic benefit of creatine. These conditions may include myopathies, muscular dystrophy, muscle wasting conditions, cancer cachexia, clinical depression, traumatic brain injuries, spinal cord injuries, orthopedic injuries, and periods of bed rest or immobilization. Lastly, several of the previously published studies which examined physical performance outcomes in healthy adult populations should be replicated in adolescents to examine if similar ergogenic benefits from creatine supplementation are possible among this population.

It is important for adolescents, coaches, and parents to be aware of evidence-based recommendations regarding the safety and efficacy of creatine supplementation when considering its use. Many misconceptions are present regarding creatine [81], and therefore consumers should seek out expert advice regarding safe and informed use of creatine. Readers are also directed to the most recently published Position Stand on creatine published by the International Society of Sports Nutrition [1] for a complete summary regarding the mechanisms of action, ergogenic benefits, safety, clinical applications and dosing recommendations of creatine. In brief, the statements from the position stand below are most pertinent to the focus of the current review article:

- “Creatine monohydrate is the most effective ergogenic nutritional supplement currently available to athletes with the intent of increasing high intensity exercise capacity and lean body mass during training.”
- “Creatine monohydrate supplementation is not only safe, but has been reported to have a number of therapeutic benefits in healthy and diseased populations ranging from infants to the elderly. There is no compelling scientific evidence that the short- or long-term use of creatine monohydrate (up to 30 g/day for 5 years) has any detrimental effects on otherwise healthy individuals or among clinical populations who may benefit from creatine supplementation.”
- “If proper precautions and supervision are provided, creatine monohydrate supplementation in children and adolescent athletes is acceptable and may provide a nutritional alternative with a favorable safety profile to potentially dangerous anabolic androgenic drugs. However, we recommend that creatine supplementation only be considered for use by younger athletes who: (a) are involved in serious/competitive supervised training; (b) are consuming a well-balanced and performance-enhancing diet; (c) are knowledgeable about the appropriate use of creatine; and (d) do not exceed recommended dosages.”
- “Label advisories on creatine products that caution against usage by those under 18 years old, while perhaps intended to insulate their manufacturers from legal liability, are likely unnecessary given the science supporting creatine’s safety, including in children and adolescents. The quickest method of increasing muscle creatine stores may be to consume ~0.3 g/kg/day of creatine monohydrate for 5–7-days followed by 3–5 g/day thereafter to maintain elevated stores. Initially, ingesting smaller amounts of creatine monohydrate (e.g., 3–5 g/day) will increase muscle creatine stores over a 3–4 week period, however, the initial performance effects of this method of supplementation are less supported.”

## Figures and Tables

**Table 1:** Efficacy of creatine use in adolescents on exercise performance.

Author Year (Country)	Subjects	Design	Duration	Dosing Protocol	Primary Variables	Results	Adverse Events
Swimming							
Dawson et al. 2002 (Australia) [36]	10 male, 10 female (16.4 ± 1.8 years) swimmers	Matched, placebo-controlled	4 weeks	20 g/day (5 days) 5 g/day (22 days)	Sprint swim performance and swim bench test	↑ swim bench test performance	None reported
Grindstaff et al. 1997 (USA) [33]	18 (11 female, 7 male) adolescent swimmers (15.3 ± 0.6 years)	Randomized, double-blind, placebo controlled	9 days	21 g/day	Sprint swim performance; arm ergometer performance	↑ sprint swimming performance	None reported
Juhasz et al. 2009 (Hungary) [34]	16 male fin swimmers (15.9 ± 1.6 years)	Randomized, placebo-controlled, single-blind trail	5 days	20 g/day	Average power, dynamic strength (swim based tests)	↑ anaerobic performance; ↑ dynamic strength	None reported
Theodorou et al. 1999 (UK) [37]	10 elite female (17.7 ± 2.0 years) and 12 elite male (17.7 ± 2.3 years) swimmers	Randomized, double-blind, placebo-controlled	11 weeks	25 g/day (4 days) 5 g/day (2 months)	Swimming interval performance	↑ interval performance following loading phase; 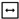 long-term improvements after maintenance dose	None reported
Theodorou et al. 2005 (United Kingtom) [35]	10 high performance swimmers (males: *n* = 6; females: *n* = 4) (17.8 ± 1.8 years	Randomized, double-blind trial	4 days	20 g/day of CrM or 20 g/day of CrM + 100 g of carbohydrates per serving	High-intensity swim performance during repeated intervals	↑ mean swim velocity for all swimmers; 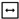 swim velocity in Cr + Carbohydrate condition	Gastrointestinal discomfort in CrM + Carbohydrate group only
Soccer							
Claudino et al. 2014 (Brazil) [42]	14 male Brazilian elite soccer players (18.3 ± 0.9 years)	Randomized, double-blind, placebo-controlled	7 weeks	20 g/day (1 week) 5 g/day (6 weeks)	Lower limb muscle power via countermovement vertical jump	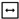 lower body power	None reported
Mohebbi et al. 2012 (Iran) [39]	17 adolescent soccer players (17.2 ± 1.4 years)	Randomized, double-blind, placebo-controlled	7 days	20 g/day	Repeated sprint test, soccer dribbling performance and shooting accuracy	↑ repeat sprint performance; ↑ dribbling performance	None reported
Ostojic et al. 2004 (Yugoslavia) [40]	20 adolescent male soccer players (16.6 ± 1.9 years)	Matched, placebo-controlled	7 days	30 g/day	Soccer specific skills tests	↑ dribble test and endurance times; ↑ sprint power test and countermovement jump	None reported
Yanez-Silva et al. 2017 (Brazil) [41]	Elite youth soccer players (17.0 ± 0.5 years)	Matched, double-blind, placebo-controlled	7 days	0.03 g/kg/day	Muscle power output (Wingate anaerobic power test)	↑ peak and mean power output; ↑ total work	None reported

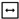
 = Creatine supplementation resulted in no significant (*p* > 0.05) change; ↑ = Creatine supplementation resulted in a significant increase (*p* < 0.05) over control. CrM = creatine monohydrate; g/day = grams per day. Adapted from Jagim et al. 2018 [43].

**Table 2:** Efficacy of creatine in clinical settings.

Author Year	Subjects	Design	Duration	Dosing Protocol	Primary Variables	Results	Adverse Events
Sipila et al. 1981 [57]	7 (3 adolescents) patients with gyrate atrophy of retina	Open label treatment intervention	12 months	1.5 g/day	Visual acuity, muscle fiber characteristics, laboratory markers of creatine metabolism	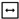 Visual acuity; ↑ Thickness of Type II muscle fibers	No side effects reported
Vannas-Sulonen et al. 1985 [58]	13 patients (9 male, 4 female) between ages of 6–31 years diagnosed with gyrate atrophy of the choroid	Prospective, open-label cohort	36–72 months	0.25–0.5 g dose 3×/day	Morphological and eye function assessments	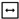 Cr supplementation did not prevent normal deterioration; ↓ Muscle atrophy, primarily in type II fibers	None reported
Walter et al. 2000 [59]	36 patients with multiple types of muscular dystrophies (overall mean age: 26 ± 16 years) 8 patients with Duchenne dystrophy (mean age: 10 ± 3 years)	Randomized, double-blind, placebo-controlled	8 weeks	10 g/day (adults) 5 g/day (children)	Muscular performance, neuromuscular symptoms score, vital capacity and qualitative assessments	↑ (3%) in muscle strength; ↑ (10%) in neurological symptoms. Children tended to experience greater strength changes.	None reported. Indicated to be well-tolerated.
Braegger et al. 2003 [60]	18 cystic fibrosis patients (7 F, 11 M) ranging in age from 8–18 years	Prospective open-label pilot	Supplemented for 12 weeks; monitored for 24–36 weeks	12 g/day for 1st week; 6 g/day for remaining 11 weeks	Lung function, strength, and clinical parameters	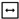 Lung function or sweat electrolytes. ↑ (18%) in peak isometric strength	One patient experienced transient muscle pain; No other side effects
Louis et al. 2003 [61]	15 boys with muscular dystrophy (mean age: 10.8 ± 2.8 years)	Double-blind, placebo-controlled, cross-over study design	3 months, with 2 months washout	3 g/day	Muscle function, densitometry, markers of hepatic and renal function, magnetic resonance spectroscopy	↑ MVC by 15% ↑ TTE (~2×) ↑ TJS ↑ LS and WB BMD in ambulatory patients ↑ NTx/creatinine ratio in ambulatory patients	No changes in liver or kidney markers
Tarnopolsky et al. 2004 [45]	30 boys with Duchenne muscular dystrophy; mean age: 10 ± 3 years; height: 129.2 ± 16.0 cm; weight: 35.3 ± 15.8 kg	Double-blind, randomized, crossover trial	4 months	0.10 g/kg/day	Pulmonary function, strength, body composition, bone health, task function, blood & urinary markers	↑ handgrip strength, fat-free mass, and bone markers 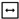 functional tasks or activities of daily living	None
Escolar et al. 2005 [49]	50 ambulatory steroid naïve boys with Duchenne Muscular Dystrophy (mean age: 6 years)	Double-blind, placebo-controlled, randomized	6 months	5 g/day of creatine powder, 0.3 mg/kg of glutamine (×2 per day), or placebo	Manual muscle performance, quantitative muscle testing, time to rise	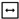 primary or secondary outcomes measures	Deemed safe and well-tolerated with no side effects reported.
Sakellaris et al. 2008 [62]	39 children/adolescents following traumatic brain injury	Open-label pilot study	6 months	0.4 g/kg/day	Duration of amnesia, duration of intubation, and intensive care unit stay post traumatic brain injury	↓ Amnesia ↓ Intubation period ↓ Intensive care unit stay	None
Bourgeois et al. 2008 [63]	9 children with lymphoblastic leukemia during chemotherapy (in treatment group); mean age of 7.6 years, 50 healthy children as history controls	Cross sectional, mixed cohort designs	16 weeks	0.1 g/kg/day	Height, weight, BMI, BMD, BMC, FFM, %BF, serum creatinine	↑ %BF and BMI	None reported
Banerjee et al. 2010 [9]	33 ambulatory male patients with Duchenne muscular dystrophy	Randomized, placebo-controlled, single-blind trial	8 weeks	Cr, 5 g/day (*n* = 18)	Cellular energetics, manual muscle test score and functional status	↑ in PCr/Pi ratios	None reported
Van de Kamp et al. 2012 [16]	9 boys with creatine transporter defect	Long-term follow-up investigation	4–6 years	Cr (400 mg/kg/day) and L-arginine (400 mg/kg/day)	Locomotor and personal social IQ subscales	Initial ↑ in locomotor and personal social IQ subscales; No lasting clinical improvement was recorded	No adverse events were reported.
Hyashi et al. 2014 [13]	15 participants with childhood systemic lupus erythematosus	Double-blind, placebo controlled, cross-over design	12 weeks with 8 week washout period	0.1 g/kg/day	Muscle function, body composition, biochemical markers of bone, aerobic conditioning, quality of life	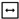 intramuscular PCr, muscle function, and aerobic conditioning parameters, body composition, quality of life	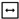 laboratory parameters; No side effects reported
Solis et al. 2016 [12]	Patients with juvenile dermatomyositis (mean age: 13 ± 4 years)	Randomized, double-blind, placebo-controlled, crossover trial	12 weeks	0.1 g/kg/day	Primary: muscle function Secondary: body composition, biochemical markers of bone remodeling, cytokines, laboratory markers of kidney function, aerobic conditioning, and quality of life	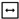 Muscle function, intramuscular PCr content, or other secondary outcomes measures	No side efforts reported. 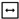 Markers of kidney function
Kalamitsou et al. 2019 [64]	22 children (9 F, 13 M) with refractory epilepsy ranging in age from 10 months to 8 years	Prospective cohort	3–12 months follow-up	0.4 g/kg/day creatine + ketogenic diet	Proportion of responders to ketogenic diet	6/22 (27%) responded to creatine addition to ketogenic diet	None reported, well-tolerated with no exacerbations of underlying pathology
Dover et al. 2020 [65]	13 (7 F, 6 M) patients ranging in age from 7–14 years with juvenile dermatomyositis; 25.6–64.6 kg; 14.3–22.9 kg/m^2^	Randomized, double-blind, placebo-controlled	6 months	Up to 40 kg was 150 mg/kg/day >40 kg was 4.69 g/m^2^/day	Safety and tolerability muscle function, disease activity, aerobic capacity, muscle strength	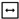 in muscle function, strength, aerobic capacity, fatigue, physical activity ↓ in muscle pH following exercise	No adverse events reported

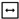
 = Creatine supplementation resulted in no change in the target outcome; ↑ = Creatine supplementation resulted in an increase in the target outcome; ↓ = Creatine supplementation resulted in a decrease (directional) in the target outcome. BMI = body mass index; FFM = fat-free mass; %BF = body fat percentage; TJS = total joint stiffness; TTE = time to exhaustion; g/d = grams per day; g/kg/d = grams per kilogram of bodyweight per day; mg/kg/d = milligrams per kilogram of bodyweight per day; PCr = phosphocreatine. MVC = maximum voluntary contraction; NTx = N-terminal telopeptide of type I collagen; LS = lumbar spine; WB = whole body; BMD = bone mineral density; BMC = bone mineral content; Pi = inorganic phosphate.

## Data Availability

Data sharing not applicable: No new data were created or analyzed in this study.
